# High neutrophil-to-lymphocyte ratios confer poor prognoses in patients with small cell lung cancer

**DOI:** 10.1186/s12885-017-3893-1

**Published:** 2017-12-21

**Authors:** Dan Liu, Yi Huang, Lei Li, Juan Song, Li Zhang, Weimin Li

**Affiliations:** 1 Department of Pulmonary & Critical Care Medicine, West China Hospital of Sichuan University, Chengdu, 610041 Sichuan Province People’s Republic of China; 2 Clinical Laboratory Department, Sichuan Academy of Medical Sciences & Sichuan Provincial People’s Hospital, Affiliated Hospital of University of Electronic Science and Technology of China, Chengdu, 610072 Sichuan Province People’s Republic of China; 3 Laboratory of Pathology, West China Hospital of Sichuan University, Chengdu, 610041 Sichuan Province People’s Republic of China

**Keywords:** Neutrophil-to-lymphocyte ratio, Platelet-to-lymphocyte ratio, Small cell lung cancer, Prognosis, Mortality risk

## Abstract

**Background:**

The neutrophil-to-lymphocyte ratio (NLR) and platelet-to-lymphocyte ratio (PLR) are easily obtained from routine blood tests. We investigated the associations of the NLR and PLR with the clinical parameters and prognoses of small cell lung cancer (SCLC) patients.

**Methods:**

Pre-treatment clinical and laboratory data from 139 patients with SCLC were retrospectively studied with univariate analyses. The NLR and PLR values were divided into two separate groups: high NLR (>4.55, *n* = 32) vs low NLR (≤4.55, *n* = 107) and high PLR (>148, *n* = 63) vs low PLR (≤148, *n* = 76). Kaplan-Meier survival analyses and Cox proportional hazard models were used to examine the effects of NLR and PLR on overall survival.

**Results:**

Chi-square analyses revealed significant associations of high NLR with tumour stage, hepatic metastasis, radiotherapy and chemotherapy and significant associations of high PLR with tumour stage, bone and hepatic metastases, exposure to cooking oil fumes, and chemotherapy. Mann-Whitney U tests demonstrated an association of high NLR with smoking exposure, and high NLR and high PLR were correlated with several laboratory parameters. Kaplan-Meier analyses revealed that high NLR and high PLR conferred poor prognoses for SCLC patients. Moreover, multivariate analysis demonstrated that NLR, tumour stage, and hepatic metastasis were independent prognostic factors for survival. In this study, we found that NLR and PLR were associated with several factors that reflect the inflammatory (white blood cell count, WBC; lactate dehydrogenase, LDH) and nutritional (albumin, ALB; haemoglobin, HB; and cholesterol) status of SCLC patients at diagnosis.

**Conclusions:**

NLR is an independent prognostic factor and can be used to predict the mortality risk of SCLC patients.

## Background

Lung cancer is the leading cause of cancer-related death worldwide, and small cell lung cancer (SCLC) accounts for 15% of lung cancers. SCLC has the most aggressive clinical course of all pulmonary tumour types with a median survival from diagnosis of only 2 to 4 months without treatment [1]. Even with treatment, the median survival time for patients with limited stage SCLC is less than 24 months, and for those with extensive stage, the median survival is no more than 12 months [1, 2]. Several clinical markers are related to the prognoses of SCLC patients, including tumour stage, sex, serum carcinoembryonic antigen (CEA) and lactate dehydrogenase (LDH), and indicate a high tumour burden and a poor prognosis [3–7]. Albumin (ALB), haemoglobin (HB), and cholesterol (CHO), which reflect nutritional status, can also be of prognostic value [8–10]. However, the optimized prognostic factors for SCLC remains controversial [11]. Inflammation of the micro-environment plays a pivotal role in the development and progression of malignancies by influencing the proliferation and survival of tumour cells, promoting angiogenesis and metastasis, and reducing responses to anti-cancer agents [12]. Platelet activation is also stimulated by proinflammatory cytokines and participates in neutrophil recruitment [13]. Recent studies have focused on the relationships of inflammatory factors, the neutrophil-to-lymphocyte ratio (NLR) and the platelet-to-lymphocyte ratio (PLR) with survival in patients with various cancer types including SCLC [2, 11, 14–21]. Meta-analyses results also highlighted the prognostic values of NLR and PLR in various solid tumours [22, 23]. This study aims to investigate the clinical significance of pre-treatment NLR and PLR values and their relationship with the overall survival of Chinese patients with SCLC.

## Methods

### Study populations

One hundred and thirty nine patients diagnosed with SCLC in the West China Hospital between January 2009 and October 2013 was retrospectively analysed. This study was approved by the institutional review board of the West China Hospital of Sichuan University. The diagnoses of SCLC were made pathologically with surgically resected specimens, bronchoscopic biopsies, or CT-guided needle lung biopsies. Blood samples were collected from patients according to the standard operating procedures at diagnosis. HB, mean corpuscular volume (MCV), red blood cell count (RBC), platelets (PLT), white blood cell count (WBC), neutrophil and lymphocyte counts were determined with XE-2100 and XE-5000 systems (Sysmex corporation, Kobe, Japan). Serum ALB, LDH, alkaline phosphates (ALP), CHO, triglycerides (TG), high-density lipoprotein cholesterol (HDL-C), low-density lipoprotein cholesterol (LDL-C) and creatinine (CR) were determined with a cobas 8000 analyser (Roche, Mannheim, Germany). Serum CEA, cytokeratin fragment antigen 21–1 (CYFRA21-1), and neuron-specific enolase (NSE) were determined with a cobas E601 system (Roche, Mannheim, Germany). The NLR was defined as the ratio of neutrophil the count to the lymphocyte count, and the PLR was defined as the ratio of the PLT to the lymphocyte count. ROC (receiver operating characteristic) curves were used to define the cutoff values for the NLR and PLR.

### Statistical analysis

Continuous variables are presented as the means ± the SDs or the medians (first quartile-third quartile). The Statistical Product and Service Solutions 17.0 software (SPSS, Chicago, IL, USA) for Windows was used to perform the statistics. Student’s t tests and Mann-Whitney U tests were used to compare normally and non-normally distributed variables, respectively. The Kaplan-Meier method was used to draw the survival rate curves, and the log-rank test was used to compare the differences in the curves. Univariate and multivariate Cox proportional hazard models were used to evaluate the prognostic variables. *p* < 0.05 was considered statistically significant.

## Results

This study included 139 SCLC patients with the average age of 58.4 years old. Among these patients, 107 were male, and 32 were female. At the time of diagnosis, 55 cases had limited disease (LD) stage, and 83 cases had extensive disease (ED) stage. Thirty-nine patients were non-smokers, and the other 100 were current or ex-smokers. The median NLR and PLR values were 3.13 and 132.7, respectively (Table 1).

**Table 1:** Basic characteristics of patients with small cell lung cancer

	Patients
No.	139
Mean age (years, ± SD)	58.4 ± 10.5
Gender (male/female)	107/32
Stage (LD/ED)	55/83
Smoking (never/ever)	39/100
NLR	3.13 (2.23–4.50)
PLR	132.7 (97.8–186.5)

The data are presented as the means ± the SD or the medians (first quartile-third quartile)

*LD* limited stage, *ED* extensive stage

### NLR values and clinical parameters

As defined by the ROC curve analyses, patients with NLR values ≤4.55 (*n* = 107) and >4.55 (*n* = 32) were classified as the low NLR and high NLR groups, respectively. The clinical and laboratory data are presented in Table 2. The high NLR group patients exhibited more advanced tumour stages (*p* = 0.005), a higher hepatic metastatic rate (*p* = 0.020), a greater amount of smoking (*p* = 0.031), and lower frequencies of patients received radiotherapy (*p* = 0.041) and chemotherapy (*p* = 0.043). These patients also exhibited higher PLR (*p* = 0.000), PLT (*p* = 0.035), WBC (*p* = 0.001), neutrophil (*p* = 0.000), lymphocyte (*p* = 0.000), LDH (*p* = 0.001), CYFRA21-1 (*p* = 0.017), and NSE (*p* = 0.034) levels and lower levels of RBC (*p* = 0.002), HB (*p* = 0.001), ALB (*p* = 0.000), CHO (*p* = 0.000), HDL-C (*p* = 0.007), and LDL-C (*p* = 0.001).

**Table 2:** Clinical and laboratory data from SCLC patients stratified by NLR

Variables	NLR ≤ 4.55		NLR > 4.55		*p* value
		*n*		*n*	
Age (years, ± SD)	57.61 ± 10.63	107	60.81 ± 9.70	32	0.129
Gender (male/female)	81/26	107	26/6	32	0.513
Stage (LD/ED)	50/57	107	6/26	32	0.005**
Metastasis (yes/no)	80/27	107	28/4	32	0.129
Brain metastasis (yes/no)	7/100	107	2/30	32	1.000
Bone metastasis (yes/no)	9/98	107	6/26	32	0.111
Liver metastasis (yes/no)	11/96	107	9/23	32	0.020*
Adrenal metastasis (yes/no)	10/97	107	4/28	32	0.738
Pleural metastasis (yes/no)	7/100	107	5/27	32	0.147
Lymph node metastasis (yes/no)	72/35	107	27/5	32	0.061
Mediastinal metastasis (yes/no)	3/104	107	1/31	32	1.000
Smoking (never/ever)	32/75	107	7/25	32	0.375
Smoking amount (BI)	660 (380–970)	73	860 (600–1200)	25	0.031*
Family cancer history (yes/no)	19/48	67	8/12	20	0.323
Exposure to cooking oil fume (never/ever)	32/31	63	7/12	19	0.286
Operation (yes/no)	12/95	107	2/30	32	0.413
Radiotherapy (yes/no)	52/55	107	9/23	32	0.041*
Chemotherapy (yes/no)	96/11	107	24/8	32	0.043*
PLR	115 (89–165)	107	221 (175–324)	32	0.000***
RBC (10^12^/L)	4.49 (4.17–4.88)	107	4.18 (3.47–4.47)	32	0.002**
Hb (g/L)	135 (124–146)	107	120 (106–134)	32	0.001**
MCV (fL)	91.4 (87.8–94.1)	107	92.2 (87.6–93.8)	32	0.906
PLT (10^9^/L)	181 (138–228)	107	206 (169–289)	32	0.035*
WBC (10^9^/L)	6.13 (5.07–7.23)	107	7.34 (6.34–9.75)	32	0.001**
Neutrophil (10^9^/L)	4.08 (3.00–4.98)	107	5.62 (4.98–7.58)	32	0.000***
Lymphocyte (10^9^/L)	1.46 (1.22–1.82)	107	0.96 (0.71–1.10)	32	0.000***
Alb (g/L)	41.2 (38.6–43.6)	104	37.1 (33.0–40.2)	32	0.000***
LDH (U/L)	193 (175–238)	102	264 (205–360)	31	0.001**
ALP (U/L)	79 (65–95)	104	82 (59–114)	32	0.595
CHO (mmol/L)	4.46 (4.06–4.90)	102	3.87 (3.30–4.47)	31	0.000***
TG (mmol/L)	1.22 (0.88–1.45)	102	1.06 (0.87–1.46)	31	0.661
HDL-C (mmol/L)	1.25 (1.08–1.54)	102	1.09 (0.94–1.34)	31	0.007**
LDL-C (mmol/L)	2.63 (2.27–3.00)	102	2.23 (1.63–2.72)	31	0.001**
CR (μmol/L)	76.1 (65.3–87.9)	104	67.6 (55.4–83.7)	32	0.082
CEA (ng/ml)	3.71 (1.80–9.13)	85	3.66 (2.04–10.25)	29	0.747
CYFRA21-1 (ng/ml)	3.10 (2.27–4.84)	75	4.76 (2.78–7.43)	26	0.017*
NSE (ng/ml)	38.85 (22.86–97.62)	86	71.29 (37.66–113.23)	26	0.034*

The data are presented as the means ± the SDs or medians (first quartile-third quartile)

*p < 0.05, **p < 0.01, ***p < 0.001

*BI* Brinkman index, which was calculated by multiplying the number of cigarettes smoked per day by the duration of smoking in years

*RBC* red blood cell

*HB* hemoglobin

*MCV* mean cell volume

*PLT* platelet

*WBC* white blood cell

*Alb* albumin

*LDH* lactate dehydrogenase

*ALP* alkaline phosphates

*CHO* cholesterol

*TG* triglyceride

*HDL-C* high density lipoprotein-cholesterol

*LDL-C* low density lipoprotein-cholesterol

*CR* creatinine

*CEA* carcinoembryomic antigen

*CYFRA21-1* cytokeratin fragment antigen 21–1

*NSE* neuron specific enolase

### PLR values and clinical parameters

Patients with PLR ≤148 (*n* = 76) and >148 (*n* = 63) were divided into the low PLR and high PLR groups, respectively. The clinical and laboratory data are presented in Table 3. The patients in the high PLR group exhibited more advanced tumour stages (*p* = 0.000), higher bone and hepatic metastases rates (*p* = 0.021 and *p* = 0.004), higher frequencies of exposure to cooking oil fumes (*p* = 0.022), and lower rates of patients received chemotherapy (*p* = 0.008). These patients also had higher NLR (*p* = 0.000), PLT (*p* = 0.000), and LDH (*p* = 0.023) levels and lower levels of RBC (*p* = 0.000), HB (*p* = 0.000), MCV (*p* = 0.012), neutrophil (*p* = 0.000), lymphocyte (*p* = 0.000), ALB (*p* = 0.000), CHO (*p* = 0.002), LDL-C (*p* = 0.003), and CR (*p* = 0.011).

**Table 3:** Clinical and laboratory data from SCLC patients stratified by PLR

Variables	PLR ≤ 148		PLR > 148		*p* value
		*n*		*n*	
Age (years, ±SD)	58.54 ± 9.60	76	58.11 ± 11.52	63	0.811
Gender (male/female)	60/16	76	47/16	63	0.552
Stage (LD/ED)	43/33	76	13/50	63	0.000***
Metastasis (yes/no)	56/20	76	52/11	63	0.212
Brain metastasis (yes/no)	3/73	76	6/57	63	0.299
Bone metastasis (yes/no)	4/72	76	11/52	63	0.021
Liver metastasis (yes/no)	5/71	76	15/48	63	0.004
Adrenal metastasis (yes/no)	6/70	76	8/55	63	0.349
Pleural metastasis (yes/no)	4/72	76	8/55	63	0.120
Lymph node metastasis (yes/no)	53/23	76	46/17	63	0.671
Mediastinal metastasis (yes/no)	2/74	76	2/61	63	1.000
Smoking (never/ever)	19/57	76	20/43	63	0.449
Smoking amount (BI)	720 (400–980)	55	680 (400–1200)	43	0.725
Family cancer history (yes/no)	16/31	47	11/29	40	0.511
Exposure to cooking fume (never/ever)	27/19	46	12/24	36	0.022*
Operation (yes/no)	11/65	76	3/60	63	0.058
Radiotherapy (yes/no)	39/37	76	22/41	63	0.052
Chemotherapy (yes/no)	71/5	76	49/14	63	0.008**
NLR	2.59 (1.69–3.17)	76	4.43 (2.88–5.50)	63	0.000***
RBC (10^12^/L)	4.57 (4.18–5.02)	76	4.24 (3.74–4.57)	63	0.000***
Hb (g/L)	139 (126–149)	76	124 (107–136)	63	0.000***
MCV (fL)	92.4 (89.1–95.7)	76	90.1 (86.4–93.3)	63	0.012*
PLT (10^9^/L)	155 (130–192)	76	226 (192–299)	63	0.000***
WBC (10^9^/L)	6.45 (5.29–7.81)	76	6.52 (5.15–7.57)	63	0.828
Neutrophil (10^9^/L)	4.11 (3.02–5.20)	76	4.64 (3.59–5.76)	63	0.075
Lymphocyte (10^9^/L)	1.63 (1.32–2.00)	76	1.08 (0.87–1.29)	63	0.000***
Alb (g/L)	41.8 (39.5–44.2)	75	38.2 (34.8–41.2)	61	0.000***
LDH (U/L)	196 (176–239)	73	219 (183–290)	60	0.023*
ALP (U/L)	79 (66–93)	75	78 (59–107)	61	0.941
CHO (mmol/L)	4.47 (4.09–4.96)	73	4.15 (3.62–4.59)	60	0.002**
TG (mmol/L)	1.27 (0.91–1.68)	73	1.07 (0.82–1.43)	60	0.115
HDL-C (mmol/L)	1.24 (1.07–1.50)	73	1.18 (0.98–1.49)	60	0.144
LDL-C (mmol/L)	2.65 (2.40–3.18)	73	2.38 (2.08–2.82)	60	0.003**
CR (μmol/L)	76.9 (70.9–89.0)	75	69.2 (59.0–83.6)	61	0.011*
CEA (ng/ml)	3.75 (1.90–8.21)	60	3.61 (1.90–10.87)	54	0.790
CYFRA21-1 (ng/ml)	3.32 (2.33–4.94)	51	3.35 (2.32–6.50)	50	0.370
NSE (ng/ml)	40.81 (19.22–82.20)	60	48.65 (28.26–127.48)	52	0.066

*p < 0.05, **p < 0.01, ***p < 0.001

Data are presented as the mean ± the SD or the median (first quartile-third quartile)

### NLR, PLR and prognosis

**Fig. 1:**
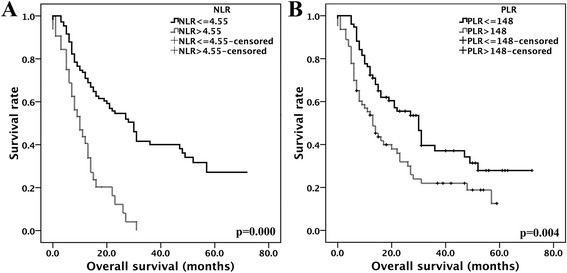
Survival analysis based on NLR and PLR. A: NLR, B: PLR

**Fig. 2:**
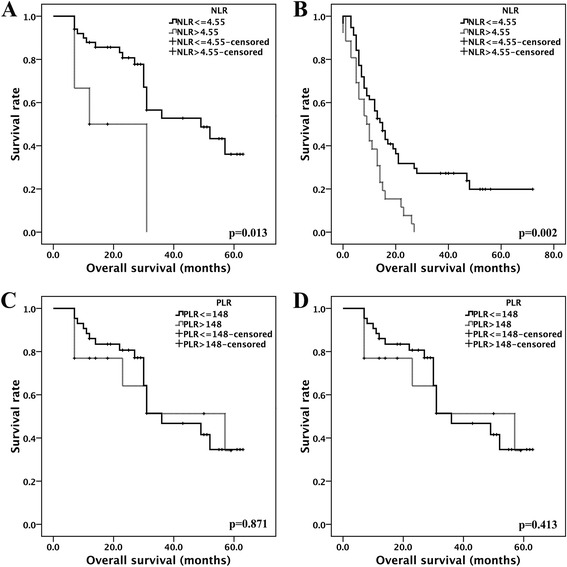
The survival functions for NLR and PLR in LD and ED patients. A: NLR in the LD group, B: NLR in the ED group, C: PLR in the LD group, D: PLR in the ED group

The overall survival times for the 139 SCLC patients were obtained by follow up study for at least 12 months. As illustrated in Fig. 1, patients in the high NLR and PLR groups exhibited worse prognoses than those with low NLR and PLR values. (log-rank tests: *p* = 0.000 and *p* = 0.004, respectively). When stratified by tumour stage, the high NLR patients in both the LD (*n* = 56) and ED (*n* = 83) stages exhibited shorter overall survival times (log-rank tests: *p* = 0.013 and *p* = 0.002, respectively; Fig. 2A-B). No significant differences were observed in the high PLR group when stratified by tumour stage (log-rank tests: *p* = 0.871 and *p* = 0.413, respectively; Fig. 2C-D, Table 4). NLR > 4.55, PLR > 148, ED stage, metastases, including liver and adrenal metastases, the lack of radio- or chemotherapy, low RBC, HB, ALB level and high serum LDH level conferred poor prognoses (all *p* < 0.05). When the variables that were identified as significant in the univariate analyses were incorporated as the co-variables in the multivariate analyses, the results demonstrated that high NLR, advanced tumour stage, and hepatic metastasis were independent factors for poor survival (hazard ratio = 2.093, 95% confidence interval 1.079–4.063, *p* = 0.029; hazard ratio = 2.692, 95% confidence interval 1.501–4.830, *p* = 0.001; and hazard ratio = 2.427, 95% confidence interval 1.341–4.395, *p* = 0.003; respectively, Table 4).

**Table 4:** Univariate and multivariate Cox proportional hazard models for survival (*n* = 139)

Variables	Univariate analysis		Multivariate analysis	
	HR (95% CI)	*p* value	HR (95% CI)	*P* value
NLR > 4.55 vs ≤4.55	3.309 (2.088–5.244)	0.000***	2.093 (1.079–4.063)	0.029*
PLR > 148 vs ≤148	1.813 (1.200–2.738)	0.005**		0.332
Stage ED vs LD	3.282 (2.042–5.272)	0.000***	2.692 (1.501–4.830)	0.001**
Metastasis (yes/no)	2.085 (1.178–3.691)	0.012*		0.697
Liver metastasis yes vs no	3.377 (2.034–5.605)	0.000***	2.427 (1.341–4.395)	0.003**
Adrenal metastasis yes vs no	2.270 (1.256–4.103)	0.007**		0.398
Radiotherapy yes vs no	0.576 (0.375–0.884)	0.012*		0.479
Chemotherapy yes vs no	0.455 (0.265–0.782)	0.004**		0.098
RBC normal vs low	0.567 (0.370–0.869)	0.009**		0.128
Haemoglobin normal vs low	0.528 (0.349–0.801)	0.003**		0.608
Albumin normal vs low	0.493 (0.289–0.840)	0.009**		0.101
Lactate dehydrogenase high vs normal	1.926 (1.259–2.947)	0.003**		0.627

*HR* hazard ratio

*CI* confidence interval

*p < 0.05, **p < 0.01, ***p < 0.001

## Discussion

Over the decades, the survival time of SCLC patients has not been prolonged with or without treatment. In this study, we reviewed the prognostic significance of NLR and PLR with clinical and laboratory markers in 139 SCLC patients. Our results revealed that high pre-treatment NLR and PLR values were associated with several clinical and laboratory markers. High NLR and PLR conferred poor overall prognoses on the SCLC patients. A high NLR value, ED stage, and hepatic metastasis were independent prognostic factors for poor outcomes in SCLC.

In our study, high pre-treatment NLR and PLR values were accompanied by an increased LDH level and decreased levels of several laboratory markers, including ALB, RBC, HB, and CHO. ALB is commonly used to represent the nutritional statuses of patients, and elevated serum ALB levels are associated with improved survival among lung cancer patients [24, 25]. HB, CHO, and LDH also exhibit significant prognostic values for lung cancer patients [7, 9, 10]. These previous findings are all consistent with our results (data not shown). Kaplan-Meier analyses demonstrated that high NLR and PLR confer poor overall survival time on patients. These findings could partly be explained by selection bias in that the patients in the high NLR and PLR groups also had high LDH and low HB, CHO, and ALB levels. However, the multivariate analyses revealed that NLR was an independent prognostic factor for poor outcomes in SCLC patients. Several studies have reported controversial results regarding the prognostic values of high NLR and PLR in terms of patient survival [2, 11, 26]. The selection of different cutoff values might have contributed to this controversy. However, in the study by Wang et al., elevated NLR was an independent prognostic factor for poor overall survival of SCLC patients in both the extensive and limited stages, which corroborated our results. In our study, we do not observe an independent prognostic value of PLR for the prediction of the clinical outcomes of SCLC patients, which is also consistent with their report [21]. The similar genetic backgrounds may explain the consistency of the prognostic value of NLR, although both studies had small sample sizes (*n* = 139 in our study and *n* = 153 in theirs). Selection bias also existed in that the high NLR group included more patients in the ED stage and more hepatic metastasis; thus, these patients had poorer prognoses. However, the multivariate analysis provided evidence that high NLR, ED stage and hepatic metastasis are independent prognostic factors for poor overall survival.

Tumour metastasis, including hepatic and adrenal metastases and the lack of chemotherapy or radiotherapy were associated with poor overall survival (Table 4). These findings are consistent with previous reports of SCLC patients with and without hepatic metastasis [21, 26, 27].

Pre-treatment NLR is an easily measurable parameter. However, several reports have employed different cutoff values when evaluating the prognostic value of NLR. Inflammation conditions and steroid treatments could be confounding factors [11]. Although several studies, including our own, have found NLR to be an independent prognostic factor for cancer survival [2, 11, 26], multi-centre research is still needed to verify the association of NLR with cancer survival.

## Limitation

For the small sample size in current study, we only performed analyses to show the association between prognosis and NLR. Further validation test should be used to verify whether NLR could predict the prognosis in patients with SCLC.

## Conclusions

High NLR and PLR confer poor prognostic values on SCLC patients. NLR is an independent prognostic factor and could be used to predict the mortality risks of SCLC patients.

## Abbreviations

ALB: Albumin
ALP: Alkaline phosphates
CEA: Carcinoembryomic antigen
CHO: Cholesterol
CR: Creatinine
ED: Extensive disease.
HB: Haemoglobin
LD: Limited disease
LDH: Lactate dehydrogenase
MCV: Mean corpuscular volume
NLR: Neutrophil-to-lymphocyte ratio
NSE: Neuron specific enolase
PLR: Platelet-to-lymphocyte ratio
PLT: Platelet
RBC: Red blood cell
SCLC: Small cell lung cancer
TG: Triglyceride
WBC: White blood cell
